# Variables Associated with Alcohol Consumption and Abstinence among Young Adults in Central China

**DOI:** 10.3390/ijerph15081675

**Published:** 2018-08-07

**Authors:** Ling Qian, Ian M. Newman, Lok-wa Yuen, Duane F. Shell, Jingdong Xu

**Affiliations:** 1 Chinese Center for Health Education (CCHE), Beijing 100011, China; qianlingzh@126.com; 2 Department of Educational Psychology, University of Nebraska-Lincoln, Lincoln, NE 68588, USA; lok-wa.yuen@huskers.unl.edu (L.-w.Y.); dshell2@unl.edu (D.F.S.); 3 Hubei Institute for Health Education, Wuhan 430079, China; hbsxjd@163.com

**Keywords:** alcohol patterns, survey, interview, frequency, quantity, abstaining, heavy drinking, high-risk drinking

## Abstract

This paper presents a descriptive analysis of data gathered by personal interviews from a multistage random sample of 1640 residents aged 18–34 years in Wuhan, China. First, alcohol drinkers and abstainers were compared based on demographic, attitude, and belief variables. Next, the drinkers from the sample were classified into four groups based on frequency-quantity of alcohol use, and the frequency-quantity groups were compared on the same variables. For Abstainers versus Drinkers, we found no difference by age or gender in this sample. Married people and people with children were more likely to be abstainers. University-educated, currently-employed individuals in mid-level jobs were more likely to abstain from alcohol. Vocational/Technical graduates, people who were currently attending college, currently unemployed and never-employed individuals were more likely to be drinkers. Abstainers also responded with less-positive attitudes and beliefs about drinking and attached more importance to reasons for not drinking compared to drinkers. When the drinking frequency-quantity groups were compared, gender differences became significant: more high-quantity drinkers were women; however the guideline for quantity for women was >1 drink at a time compared to >2 drinks at a time for men. Quantity and frequency of drinking was significantly associated with having children, educational level, employment status, and type of occupation. Age, marital status, and being in college did not relate significantly with quantity and frequency of drinking alcohol. Attitudes and beliefs about drinking tended to be more positive among high-frequency and high-quantity drinkers. Drinkers in all frequency-quantity groups attached greater importance to social reasons for drinking compared to personal/psychological reasons for drinking. Drinkers in the lowest frequency-quantity group attached the most importance to reasons for not drinking. These findings confirmed that in China drinking plays an important role in socializing and celebrating, and that there are important differences between alcohol drinkers and abstainers and between frequency/quantity groups of drinkers. Western models of individualized motivation of behaviors may not accurately explain alcohol use in China. We believe the findings from this study suggest the need for more detailed studies of alcohol drinking and abstaining.

## 1. Introduction

There is limited data available on the drinking patterns of specific age groups in China. The available literature has described the socio-demographic characteristics of alcohol use [1,2,3,4,5], attitudes toward alcohol drinking [5,6,7], drinking motives [1,8,9], family factors related to drinking [1], peer alcohol use [1,5], drinking situations [1,7], health status [6,10], other substance use [2,5,6], and mental health problems [11,12]. Little attention has been paid to young adults as a group.

Descriptions of alcohol use among young people have typically been limited to student populations, and have overlooked young adults in general [13,14]. This is the first known attempt to describe the drinking characteristics of young Chinese adults ages 18 to 34 years. The data for this analysis were originally gathered as part of a four-country alcohol consumption patterns and behaviors study among young adults aged 18 to 34 by Taylor et al. [15]. Taylor et al.’s results illustrated the major differences in alcohol consumption patterns among young adults in Nigeria, Uruguay, Russia, and China. For example, the proportion of young adults who “ever drank” alcohol was 33.3% (Ilorin, Nigeria), 53.4% (Wuhan, China), 86.1% (Moscow, Russia), and 96.4% (Montevideo, Uruguay); the proportion of the sample who were abstainers was 79.8%, 54.2%, 23.1%, and 12.3% respectively. There is a need to look at the individual country sample data more closely. This paper presents a detailed analysis of the data from the China sample to see what we can learn about alcohol use and abstinence among 18–34 year olds. The results are presented in two parts. Part 1 describes the differences and similarities between drinkers and abstainers, and Part 2 explores the differences among current drinkers based on the quantity and frequency of drinking. These results will be of interest to international readers who are interested in alcohol survey methods and questions for use in China studies, and for those interested in the development of nuanced policy and education to reduce alcohol consumption. China’s population represents 19% percent of the world’s population and deserves careful attention so that proposed international alcohol policies might be effective in regions of the world outside the EU and USA. 

In 2010, in China, it was estimated that 58.4% of the males and 28.9% of the females, aged 15 years and older had consumed alcohol in the past 12 months [16]. Among these drinkers the estimated per capita consumption for males aged 15 and older was 18.7 L of pure alcohol and for females 7.6 L [16]. The prevalence of personal alcohol-related, medically-defined conditions among males was estimated to be 4.4% for alcohol dependence, 4.0% for alcohol abuse, and 10.1% for alcohol use disorder [11]. The risk of developing AUD peaked at age 30 [12].

Chinese alcohol culture shares many common characteristics with other alcohol-consuming cultures, but it also differs in important ways. Chinese traditionally view alcohol drinking as an important tool for social networking and emotional expression. Business meetings, social events, and special celebrations like weddings, funerals, and festivals are often accompanied by drinking alcohol [17]. Moderate drinking is common. Nevertheless, certain drinking customs create significant risks. For example gatherings of friends and colleagues often involve urging others to drink to establish rapport and confirm relationships [17,18]. Toasting is a way to show respect to others. Guests usually feel obligated to drink as much as the host demands. These culturally-respected social pressures make refusing to drink very difficult. In work situations younger employees are sometimes asked to drink for their supervisors and other administrators. Being capable of drinking a lot is seen as a networking ability [1], however acting badly as a result of drinking is a cause for shame [19].

## 2. Materials and Methods

### 2.1. The Data

The data used in this secondary analysis were derived from a multistage random sample of residents aged 18–34 in Wuhan, China. Wuhan is composed of 13 administrative districts. The sample size for each administrative district was proportional to the population size. Within each administrative district, communities were randomly selected, followed by random household selection. A household member aged 18–34 who had lived in the city for at least 6 months was eligible for interview. If more than one household member was eligible, the person with the most recent birthday was selected. No replacement was allowed for non-respondents. A sample size of 1600 was planned; 1640 interviews were completed [15]. 

To reduce potential bias and ensure the results accurately reflected the population of interest, the data were weighted by administrative district (urban vs. sub-urban area), age (18 to 34 years), gender, and probability of selection in the household. 

### 2.2. The Survey Instrument

The survey instrument was developed by a multinational team and collected information about alcohol use, drinking effects, drinking motives, and perception of alcohol drinking. The questionnaire used in China was developed in English, forward translated to Chinese and back translated to English. Modifications accommodated cultural and linguistic nuances. It was pretested with a small sample in Wuhan and further refined. The final questionnaire was administered in face-to-face interviews conducted by specially trained epidemiology graduate students. Interviews lasted about 15 min and were conducted in environments where respondents felt comfortable.

### 2.3. Classification of Drinkers, Abstainers, and Drinking Frequency-Quantity Groups

#### 2.3.1. Drinkers and Abstainers

Respondents were divided into drinkers and abstainers based on their answers to three alcohol consumption questions: (1) had ever consumed alcohol (excluding sips), (2) during the last 12 months how often had consumed alcohol, even a small amount, and (3) during the past 12 months, how many drinks on a typical day. Drinkers (*n* = 704) were respondents who drank alcohol in the past 12 months and consumed at least one standard drink. Abstainers (*n* = 763) were respondents who have never drunk any alcohol. To distinguish clearly between drinkers and abstainers, respondents who had ever consumed alcohol but not in the last 12 months (*n* = 122) and respondents who consumed in the past 12 months but less than one standard drink (*n* = 47) were excluded from this study. These former drinkers and occasional drinkers were too few in number to justify inclusion, and the survey provided no information on their drinking histories. Four individuals who did not provide complete drinking information were also excluded from the study. The comparison of drinkers and abstainers is presented in Part 1.

#### 2.3.2. Drinking Frequency-Quantity Groups

To understand more about the drinkers, they were further divided into groups based on frequency and quantity of drinking. Drinkers who drank at least once a month were labeled high-frequency drinkers and those who drank less than once a month were labeled low-frequency drinkers. Our quantity measure was based on Wang, Lay, Yu and Shen’s [20] discussion of the Dietary Guidelines for Chinese Residents that defined moderate alcohol consumption for women as less than 15 g of pure alcohol per day and for men as less than 25 g per day. Since one standard drink contains 10 g of alcohol in this study, drinkers were considered high-quantity if they consumed more than two drinks at a time for males and more than one drink at a time for females.

Using these criteria, four drinking groups were created: low-frequency-low-quantity (LoF-LoQ) drink less than once a month and no more than the 1–2 drinks at a time as recommended for moderate alcohol consumption; high-frequency-low-quantity (HiF-LoQ) drink more than once a month but not more than the 1–2 drink guideline; low-frequency-high-quantity (LoF-HiQ) drink less than once a month but more than the recommended 1–2 drinks when they did drink; and high-frequency-high-quantity (HiF-HiQ) drink at least once a month and more than the 1–2 drinks recommended. Comparison among the four different drinking groups is presented in Part 2.

### 2.4. Variables in Part 1

Part 1 of this analysis compared drinkers and abstainers on four variables: demographics, perceptions of drinking, acceptance of drinking, and reasons for not drinking. 

#### 2.4.1. Demographic Information

Demographic information included age, gender, marital status, highest education level attained, employment status, occupation, if a student or not, whether or not the interviewee had children.

#### 2.4.2. Perceptions of Drinking

Perceptions of drinking were assessed by three items on a 5-point bipolar scale from strongly disagree to strongly agree. They were (1) drinking is one of life’s pleasures, (2) drinking with someone else is a way of showing friendship, and (3) drinking has no benefits. With the value of skewness and excess kurtosis between −1 and +1 for all items, each item was considered as normally distributed and treated as a continuous variable, so a higher score indicated a more positive attitude towards alcohol. De Winter and Dodou [21] have concluded that a *t*-test and a Mann-Whitney-Wilcoxon test for five-point Likert items generally yielded the same results and had similar power. 

#### 2.4.3. Acceptance of Drinking

Acceptance of drinking was assessed in response to eight gender-specific drinking situations on a 5-point scale (should not drink, can drink a little but without any real effects, can drink and feel some effects but not get intoxicated, getting intoxicated is sometimes ok, and getting intoxicated is always ok). For measurement parsimony, principal component analysis, using orthogonal and oblique rotations, was conducted to create a smaller set of components. An oblique rotation provided the best defined 4-component structure with loadings above 0.3 (Table S1). The components were: (1) With Young Children (2 items: a mother spending time with young children, and a father spending time with young children), (2) Adult Private Context (2 items: a man having dinner with partner/spouse/cohabitant at home, and a woman having dinner with partner/spouse/cohabitant at home), (3) Social Drinking for Males (2 items: a man at a bar with friends, and a man out with colleagues), and (4) Social Drinking for Females (2 items: a woman out with colleagues, and a woman at a bar with friends). The components were statistically correlated ranging from −0.555 to 0.517. Internal consistency was examined using Cronbach’s alpha −0.871 for With Young Children, 0.927 for Adult Private Context, 0.777 for Social Drinking for Males, and 0.831 for Social Drinking for Females. Component scores were computed as the mean of the items in each of the component. A higher mean score indicated a greater acceptance of alcohol drinking. 

#### 2.4.4. Reasons for Not Drinking

Respondents were asked to rate the importance of 13 reasons for not drinking on a 4-point scale (not at all important, not very important, important, very important). A factor analysis failed to identify a coherent factor structure from the 13 scores, so cluster analysis was used to reduce the number of variables and identify membership groups. Three clusters emerged (Table S2). A low-rating group thought most of the reasons for not drinking were not at all important or unimportant, a high-rating group thought that most of the reasons for not drinking were important or very important, and a mid-range group whose scores were in between. The clusters suggested that respondents tended to rate all the reasons for not drinking as unimportant or all the reasons for not drinking as important. Only the mid-range cluster appeared to attribute different levels of importance to the different reasons for not drinking. 

### 2.5. Additional Variables in Part 2

Part 2 of this analysis was among the four drinking frequency-quantity groups. In addition to demographic variables, perceptions of drinking, acceptance of drinking, and reasons for not drinking, comparisons were made on the effects of drinking as well as reasons for drinking. Alcohol use in different drinking locations/situations is presented as an introduction to Part 2.

#### 2.5.1. Drinking Locations/Situations

Drinkers were asked to identify in which of seven locations/situations they drank alcohol: (1) drinking in restaurants for dinner, (2) in restaurants for lunch, (3) in bars, (4) at friends’ homes, (5) at their own home with friends, (6) at their own home alone, and (7) in public places like parks.

#### 2.5.2. Effects of Drinking

Drinkers were asked to report on 13 possible effects alcohol had on them. Answers were on a 5-point scale (never, seldom, sometimes, usually, and always). Principal component analysis, using orthogonal and oblique rotations, was conducted to create a smaller set of components. Two items were removed because they loaded on multiple components. The remaining items loaded above 0.3 resulting in a 4-component solution (Table S3). An oblique rotation provided a better defined structure. The four components were: (1) Coping (three items related to regulation of negative emotions; e.g., feel it is easier to talk about feelings), (2) Mood Enhancer (three items related to positive emotions; e.g., feel happy), (3) Problem Behavior (three items related to behaviors resulting in negative effects; e.g., conflicts with police), and (4) Sex (two items related to sex life; e.g., feel more attractive). The components were statistically correlated ranging from 0.200 to 0.515. Internal consistency for components was examined using Cronbach’s alpha −0.839 for Coping, 0.822 for Mood Enhancer, 0.620 for Problem Behavior, and 0.774 for Sex. Although the index of reliability would be relatively low for short scales, they could possess acceptable psychometric quality [22]. Component scores were computed by taking the mean score of the items in each component.

#### 2.5.3. Reasons for Drinking

Drinkers were asked to rate the importance of 11 reasons for drinking alcohol on a 4-point scale (not at all important, not very important, important, very important). Principal component analysis, using orthogonal and oblique rotations, was conducted to create a smaller set of components. The selection of items with loadings above 0.3 resulted in a 3-component solution (Table S4). An oblique rotation defined a more distinct structure. The components were: (1) Psychological Reasons (four items related to emotions; e.g., to help you relax), (2) Social Reasons (four items related to the social drinking environment; e.g., because others are drinking), and (3) Thirst/Taste (two items: because of thirst, and enjoy the taste). One item was omitted because of poor fit. The components were statistically correlated ranging from 0.192 to 0.400. Internal consistency was examined using Cronbach’s alpha −0.881 for Psychological Reasons, 0.694 for Social Reasons, and 0.591 for Thirst/Taste. Although the alpha for Thirst/Taste was lower than optimal, the scale has only two items. With two-item scales, common reliability estimators like Cronbach’s Alpha may underestimate the true reliability of the scale (see discussion in [22]). Component scores were computed as the mean of the items in each component.

### 2.6. Statistical Analysis

For the comparisons between drinkers and abstainers (Part 1), means and standard deviation for the various scales were calculated. *t*-tests and adjusted *t*-tests (if the assumption of homogeneity of variance was not met) were used to test the group mean differences. Contingency tables were created to display the frequency distribution of the categorical variables by drinking status. Chi-square tests of association were used to test the relationship between categorical variables. Effect sizes for all of these tests were calculated—Cohen’s *d* for *t*-tests and Cramer’s *V* for chi-squares.

For the comparisons between the different drinking groups (Part 2), means and standard deviations were calculated. One-way analysis of variance (ANOVA) with post hoc multiple comparisons were used to test the significance of group mean differences. When the assumption of homogeneity of variance was not met, Welch’s adjusted *F* ratio was used. Contingency tables were created to display the frequency distribution of the categorical variables by four drinking groups. Chi-square tests of association were used to test the relationship between categorical variables. Effect sizes for all tests were calculated—*f* for ANOVA and Cramer’s *V* for chi-square tests.

Data were analyzed using Statistical Package for the Social Sciences (SPSS) version 23 for Windows (SPSS Inc., Chicago, IL, USA).

## 3. Results

### 3.1. Part 1: Comparisons between Drinkers and Abstainers

#### 3.1.1. Demographic Variables of Drinkers Compared to Abstainers

Of the 1640 interviews completed, 1467 (84.5%) were valid and used in this study; 48% from drinkers and 52% from abstainers. The description of the sample is shown in Table 1. Chi-square tests were performed to test the relationship between drinking status and each demographic variable. Abstainers were more likely to be married, to have children, have a university education, or be associate-professionals or service workers. There were no drinking behavior differences by gender or age.

#### 3.1.2. Perceptions of Drinking of Drinkers Compared to Abstainers

*T*-tests were used to test the group mean differences on each item. Drinkers and abstainers viewed drinking alcohol differently. Drinkers supported the relatively positive statements about alcohol—that drinking is one of life’s pleasures and drinking with someone is a way of showing your friendship. Abstainers believed drinking had no benefits (Table 2).

#### 3.1.3. Acceptance of Drinking of Drinkers Compared to Abstainers

*T*-tests were also used to test the component mean differences between drinkers and abstainers. Significant *t* values indicate that abstainers, on average, rated the acceptance of drinking to be lower than drinkers for each drinking situation (Table 2).

#### 3.1.4. Reasons for Not Drinking of Drinkers Compared to Abstainers

A chi-square test of association shows that respondents who thought the given reasons were important were more likely to be abstainers (Table 3).

### 3.2. Part 2: Comparisons among Four Drinking Frequency-Quantity Groups

#### 3.2.1. Usual Drinking Place of Drinkers in the Sample

Drinking behavior is usually shaped by the drinking environment. In the past 12 months, more than 70% of the drinkers reported drinking at restaurants for dinner, at a friends’ homes with friends, and at home with friends. Thirty percent drank at bars, and only 14% of drinkers reported drinking with friends in public places like a park.

#### 3.2.2. Demographic Variables Associated with Drinking Frequency and Quantity

Among drinkers, 28.7% were in the LoF-LoQ group, 23.7% were in the HiF-LoQ group, 19.6% were in the LoF-HiQ group, and the remaining 28% were in the HiF-HiQ group. The demographic description of those drinking groups is presented in Table 4. Chi-square tests were used to test the demographic differences among drinker groups. Drinking behavior was related to gender, whether or not they had children, their highest education level, employment status, and occupation. Male drinkers were more likely to be in the LoF-LoQ or HiF-LoQ groups, whereas female drinkers were more likely to be in the LoF-HiQ or HiF-HiQ groups. Drinkers who had children were more likely to be HiF-HiQ drinkers, whereas drinkers who were not parents were more likely to be LoF-LoQ drinkers. Drinkers who received secondary school education or less were more likely to be in the HiF-LoQ group. In contrast, higher educated drinkers were more likely to be LoF-LoQ drinkers. Unemployed drinkers were more likely to be in the LoF-LoQ group than employed drinkers. Professionals and service workers were more likely to be HiF-HiQ drinkers, associate professionals were likely to be HiF-LoQ drinkers, and technicians and never employed people were likely to be LoF-LoQ drinkers.

#### 3.2.3. Perception of Drinking Associated with Drinking Frequency and Quantity

One-way ANOVAs with post-hoc multiple comparisons were used to test the significance of group mean differences. We found that the perceptions of drinking differed by group drinking frequency (Table 5). HiF-LoQ drinkers were more likely than LoF-LoQ drinkers to see drinking as one of life’s pleasures and to see drinking with someone as a way to show friendship. Similarly, HiF-HiQ drinkers were more likely than LoF-HiQ drinkers to agree that drinking is one of life’s pleasures and a way of showing friendship. There was no difference between drinking groups on the statement that drinking had no benefits.

#### 3.2.4. Acceptance of Drinking Associated with Drinking Frequency and Quantity 

Table 5 shows results from one-way ANOVAs with post-hoc multiple comparisons performed to examine whether the component mean scores on the acceptance of drinking in various situations differed between drinking groups. Acceptance of drinking varied within drinkers in different drinking situations. When spending time with young children, HiF-LoQ drinkers were more likely to approve of drinking than LoF-LoQ and LoF-HiQ drinkers, but HiF-HiQ drinkers had a similar degree of approval as LoF-LoQ and LoF-HiQ drinkers. During social drinking, HiF-HiQ drinkers reported higher acceptance for both male and female drinking. All drinkers felt that higher alcohol consumption was more acceptable for men than for women.

#### 3.2.5. Effects of Drinking Associated with Drinking Frequency and Quantity 

Table 6 shows results of one-way ANOVAs with post-hoc multiple comparisons of the effects of drinking by drinking groups. LoF-LoQ drinkers consistently had lower mean scores on the effects of drinking scale than HiF-HiQ drinkers. In addition, positive effects from drinking were more frequently identified than negative effects by all drinkers.

#### 3.2.6. Reasons for Drinking Associated with Drinking Frequency and Quantity

Table 6 shows the results of one-way ANOVAs with post-hoc multiple comparisons used to test group differences in reasons for drinking. The LoF-LoQ drinkers consistently gave lower scores for importance on all of the reasons for drinking compared to the HiF-HiQ drinkers. Members of all four drinking groups rated Social Reasons for drinking more important than Psychological Reasons and more important than Thirst/Taste.

#### 3.2.7. Reasons for Not Drinking Associated with Drinking Frequency and Quantity

A non-significant chi-square indicates that drinkers’ rating of the reasons for not drinking did not differ across drinking groups (Table 7).

## 4. Discussion

This paper is based on data from a community sample of young people aged 18–34 in the city of Wuhan, China. With probability sampling of a wider age range of young people, this paper compliments previous studies of young people in China, which were largely limited to samples of higher education students [13,14]. Since there is no prior data on drinking in this age group, there really is no way to know in advance what the possible confounders would be. Our goal was to shed some light on drinking patterns and beliefs that would need to be considered in future research. Some of the findings from this study are not consistent with earlier studies reported in the published literature. For a comparison of this sample of 18–34 year olds in Wuhan, China to with the same-age group from three other countries, see Taylor et al. [15].

This analysis of data from Chinese young adults found that gender and age (18–34 years) were not associated with whether someone drank or not. Literature on alcohol use in China has consistently reported higher drinking for males compared to females (e.g., [1,6,7,9]). Another common observation has been drinking rates increasing with age, reaching a peak in middle age [2,10]. Observations by our research team in China suggest that it is becoming increasingly acceptable for females to admit to drinking, perhaps leading to similar rates of drinking for men and women in this survey compared to earlier studies. Women are also increasingly a part of the employed workforce, which exposes them to workplace drinking practices. 

While there was no gender difference in drinking versus abstaining, gender differences were identified in the Frequency-Quantity group analysis. Men were more likely than women to be in the low-quantity groups (LoF-LoQ and HiF-LoQ). This finding contradicted our observations of drinking culture in China, where men are often forced to drink competitively and women are not. It is possible that this survey has detected changing drinking patterns related to gender and suggests that previous generalizations based on only gender and drinking/nondrinking categories may conceal differences in quantity and frequency of drinking that are important for a better understanding of drinking behavior. We based our classifications of drinking quantity on the official Dietary Guidelines for Chinese Residents, which defined “moderate drinking” as no more than two drinks at one time for males and no more than one drink at one time for females. The different cut-off points explains why the majority of the 18- to-34-year-old females in this survey were in the high-quantity groups (LoF-HiQ and HiF-HiQ) and the majority of the 18- to 34-year old males were in the low-quantity groups (LoF-LoQ and HiF-LoQ). A different result would have come from using a different definition for moderate drinking. We acknowledge that the drinking definitions used are conservative by Western standards, but they are consistent with Chinese drinking culture. Taylor et al.’s four-country study [15] found a comparatively low rate of excessive drinking for the China sample compared to the other three country samples. It is also important to keep in mind that 40% of the China sample reported drinking spirits, which in China range in strength from 40% to 60% alcohol by volume.

The 18- to 34-year-olds with vocational/professional (non-university) education, as a group, had the fewest abstainers compared to those who had university degrees or those who had secondary or less education. Wu and colleagues [4] report that in China higher education levels were related to lower drinking rates, while others indicate the opposite [2,3]. Previous studies and our results do not present a clear picture of the relationship between education and drinking. 

We found that professionals and technicians were more likely to be drinkers than associate professionals and service workers. This is consistent with results reported by Li and colleagues [3] who suggested higher drinking rates for workers in the more technical manufacturing and construction industry than those in the less technical service sector. Assuming more education is required for the more technical occupations, this finding appears to contradict earlier studies that found higher levels of education associated with lower levels of drinking. Wu and colleagues [4] found no significant difference in the proportion of regular alcohol drinkers across different occupations among rural or urban respondents. Unfortunately different occupation categories were used in the different studies, and not all survey-designed occupational categories correlate to education levels. Thus, a unified set of occupation categories is needed to truly assess the relationship between occupation, education, and drinking behavior.

The present study indicated that married respondents and respondents who had a child were more likely to be abstainers. This finding contrasts with findings reported in earlier Chinese literature which reported that being married was associated with higher drinking rates [2,3,4]. Married couples planning to start a family may be benefitting from the increase in community health information about the risks of alcohol on pregnancy, especially among the higher educated Chinese. Married people with children may have benefited from clinic-based education services associated with childbirth and well-baby clinics. Interestingly, even though people with children were more likely to be abstainers, the drinkers with children were more likely to fall into the HiF-HiQ category, especially those employed as professionals, service workers, and technicians. It suggests that young people with children are also employed in occupations that require business drinking and toasting for job protection and promotion [23]. At workplace banquets and celebrations, subordinates are expected to toast their superiors, often more than once. Emptying the entire glass of alcohol indicates the highest respect during a toast. The result of toasting etiquette is both heavy and frequent drinking.

In addition to the demographic differences among the four drinking frequency-quantity groups, drinking experience seemed to influence drinking attitudes and motives. The present study consistently found that drinkers were more likely to agree that drinking is one of life’s pleasures and that drinking with someone is a way of showing friendship. Drinkers were more likely than abstainers to approve alcohol use in all four situations studied—where young children are present, in adult private context, in social drinking for men, and in social drinking for women. Frequent drinkers (HiF-LoQ and HiF-HiQ), in particular, had even stronger positive feelings about alcohol use than non-frequent drinkers (LoF-LoQ and LoF-HiQ). Positive attitudes were associated with both increased frequency of drinking and higher acceptance of others’ drinking. Other researchers have drawn this same conclusion. Wang and colleagues [1] reported that medical students with more positive attitudes toward alcohol drinking were more likely to consume alcohol. Luo and colleagues [7] noted that drinkers were more likely to approve others’ drinking behavior and drunkenness behavior than abstainers. Among college students in Qinghai, drinkers were less likely to have negative feeling about women drinking [6]. Even though drinkers were more accepting of other people’s drinking than abstainers, both drinkers and abstainers thought that drinking was not acceptable when parents were with their young children. Both groups rated social drinking among females less acceptable than social drinking among males and drinking in an adult private context, such as with a spouse, partner, or cohabitant.

The present study indicated that social motives were more important than psychological motives for drinking. Studies in China consistently reported that needs of social communication, celebrations, and coping were commonly-given reasons for drinking among college students [1,8,9]. The importance of drinking together to reinforce friendships and social networks is deeply grounded in traditional Chinese culture. 

In this study, the high-frequency drinkers (HiF-LoQ and HiF-HiQ) were more likely to report coping with negative emotions was an effect of their drinking alcohol compared to low-frequency drinkers (LoF-LoQ and LoF-HiQ). The combination of social obligations to drink and expecting positive outcomes like mood enhancement reinforces drinkers and may lead them to drink more frequently and in higher quantities.

Drinkers who participated in this survey reported the majority of their drinking was at restaurants for dinner, at a friends’ homes with friends, and at home with friends—environments that include other people. Social reasons appeared to be the main reason for drinking for all four drinking behavior groups. These findings confirm that in China drinking plays an important role in socializing, and that celebrations and festivities are typically accompanied with alcohol. Much drinking behavior research in the West is based on models and theories of behavior change that assume individualized decision-making about drinking based on experiences and outcome expectations. Clearly this is not the only way to view and understand alcohol use. Meier, Warde and Holmes [24] recognize the limitations of these individualized models and theories, and they suggest social practice theory as a complementary approach that studies drinking in the context of activities that preceded the drinking event and those that followed and the encompassing physical and social circumstances. Rather than seeing alcohol is a unitary behavior they suggest it be viewed as a practice occurring within a much larger context that incorporated cultural tradition, local values, the social and the physical environment and the expectations of what follows the drinking event. We totally agree and believe the findings from this study, especially those that do not concur with previous findings, suggest the need for alternative ways of viewing alcohol drinking.

### Limitations

This study is limited to one city: Wuhan. How similar to other cities of the same size is unknown. The data are cross-sectional in nature, thus causal inferences are not suggested. The fact that 90% of the sample reported education beyond secondary school suggests this may be an atypical city. While this study explored a variety of dimensions of drinking it asked few questions about important drinking contexts such as the workplace. This analysis is based on self-reported alcohol use/nonuse, which could be unreliable. To improve reliability the data were gathered in face-to-face interviews to reduce misunderstandings. Also, in China, there is less cultural stigma and fewer legal prohibitions on alcohol use, compared to western countries, so there was less reason for participants to underreport alcohol use. The questions on the scales used were unique to this study so comparisons are not possible. While the sample size was large, it was not large enough to explore in detail the role of the possible covariates or additional factors because the cell sizes became too small, leading to a loss of statistical power and making interpretations tenuous. Based on these results and results from other studies, future studies could be built around hypotheses of causal relationships allowing the exploration of confounding variables. Nevertheless this study did provide useful information about alcohol drinking and abstaining in the population of 18- to 34-year olds (not just students) and did use measures worthy of further refinement for future studies.

Former drinkers were excluded in this study due to small sample size and lack of information about drinking history. Former drinkers are a special group who had experience in drinking but currently do not drink. They may have a more similar attitude toward drinking to current drinkers than to lifetime abstainers. It would be worthwhile to examine the similarities and differences between former drinkers and current drinkers and lifetime abstainers in future studies.

## 5. Conclusions

These findings of this descriptive study confirm that in China drinking plays an important role in socializing, and that celebrations and festivities are typically accompanied with alcohol. Western models of individualized motivation of behaviors may not accurately explain alcohol use in China that emphasize collective values and relationships. The questions and methods used generated useful data and should be reviewed and possibly revised for use with other samples from communities from across China. Larger samples would accommodate more sophisticated analyses. This would generate a more representative picture of drinking patterns in this age group. The findings from the study, especially those that do not concur with previous findings, suggest the need for further study.

## Figures and Tables

**Table 1:** Demographic comparison of Drinkers and Abstainers.

	Drinkers		Abstainers		χ^2^	Cramer’s *V*
	*n*	%	*n*	%		
**Gender**					1.45	0.031
Male	352	46.50	405	53.50		
Female	352	49.65	357	50.35		
**Age**					0.14	0.010
18–24 years	370	48.24	397	51.76		
25–29 years	178	47.21	199	52.79		
30–34 years	156	48.45	166	51.55		
**Marital status**					9.49 *	0.081
Married	293	43.99	373	56.01		
Divorced/separated/widowed	6	75.00	2	25.00		
Never married	402	51.02	386	48.98		
**Have any children?**					9.68 *	0.082
Yes	233	43.15	307	56.85		
No	465	51.61	436	48.39		
**Education**					9.17 *	0.079
University degree or higher	255	44.58	317	55.42		
Vocational/professional/non-university tertiary education	389	51.73	363	48.27		
Secondary school or less	56	41.48	79	58.52		
**Currently a student?**					6.60 *	0.067
Yes	149	54.98	122	45.02		
No	551	46.34	638	53.66		
**Employment status**					9.36 *	0.080
Employed	490	45.97	576	54.03		
Unemployed	162	56.06	127	43.94		
Other	48	46.60	55	53.40		
**Occupation**					12.00 *	0.095
Professionals	204	50.50	200	49.50		
Associate professionals	98	43.95	125	56.05		
Service workers	190	44.71	235	55.29		
Technicians	55	52.38	50	47.62		
Never employed	109	58.29	78	41.71		

* *p* < 0.05.

**Table 2:** Perception of drinking and acceptance of drinking by drinking status.

	Drinkers		Abstainers		*t*	Cohen’s *d*
	Mean	SD	Mean	SD		
**Perception of drinking**						
*Item*						
One of life’s pleasures	3.37	1.04	2.92	0.99	−8.50 **	0.448
Drinking with someone is a way of showing friendship	3.89	0.78	3.28	0.98	−13.25 **	0.706
Drinking has no benefits	3.28	1.01	3.65	0.92	7.43 **	0.396
**Acceptance of drinking**						
*Component*						
With Young Children	1.55	0.64	1.35	0.61	−5.77 **	0.313
Adult Private Context	2.29	0.76	2.07	0.70	−5.54 **	0.304
Social Drinking for Males	2.38	0.62	2.11	0.63	−8.20 **	0.437
Social Drinking for Females	2.08	0.61	1.86	0.68	−6.40 **	0.343

*Notes*: Perception of drinking scale: 1 = strongly disagree, 5 = strongly agree; Acceptance of drinking scale: 1 = should not drink; 2 = can drink a little but without any real effects, 3 = can drink and feel some effects but not get intoxicated, 4 = getting intoxicated sometimes is OK, 5 = getting intoxicated is always OK. ** *p* < 0.001.

**Table 3:** Reasons for not drinking by drinking status.

*Cluster*	Drinkers		Abstainers		χ^2^	Cramer’s *V*
	*n*	%	*n*	%		
					73.54 **	0.257
Most reasons for not drinking are:						
Not very important–unimportant (low-rating group)	211	60.98	135	39.02		
Importance varied by reason (mid-range group)	260	57.78	190	42.22		
Important–very important (high-rating group)	97	30.79	218	69.21		

** *p* < 0.001.

**Table 4:** Demographic descriptions by drinking frequency-quantity groups.

	Low Frequency-Low Quantity (LoF-LoQ)		High Frequency-Low Quantity (HiF-LoQ)		Low Frequency-High Quantity (LoF-HiQ)		High Frequency-High Quantity (HiF-HiQ)		χ^2^	Cramer’s *V*
	*n*	%	*n*	%	*n*	%	*n*	%		
**Gender**									58.58 **	0.289
Male	117	33.24	113	32.10	37	10.51	85	24.15		
Female	85	24.22	54	15.39	100	28.49	112	31.91		
**Age**									4.09	0.054
18–24 years	117	31.54	87	23.45	71	19.14	96	25.88		
25–29 years	44	25.00	42	23.86	34	19.32	56	31.82		
30–34 years	40	25.81	38	24.52	32	20.65	45	29.03		
**Marital status**									5.27	0.087
Married	71	24.40	70	24.05	60	20.62	90	30.93		
Never married	128	32.00	93	23.25	76	19.00	103	25.75		
**Have any children?**									10.31 *	0.122
Yes	49	21.21	56	24.24	52	22.51	74	32.03		
No	150	32.26	111	23.87	85	18.28	119	25.59		
**Education**									13.31 *	0.098
University degree or higher	75	29.64	58	22.92	51	20.16	69	27.27		
Vocational/professional/non-university tertiary education	118	30.49	86	22.22	75	19.38	108	27.91		
Secondary school or less	7	12.50	22	39.29	8	14.29	19	33.93		
**Currently a student?**									4.72	0.082
Yes	49	32.89	31	20.81	34	22.82	35	23.49		
No	148	26.96	136	24.77	103	18.76	162	29.51		
**Employment status**									15.48 *	0.106
Employed	128	26.28	132	27.10	90	18.48	137	28.13		
Unemployed	59	36.42	27	16.67	34	20.99	42	25.93		
Other	10	21.74	7	15.22	13	28.26	16	34.78		
**Occupation**									35.44 **	0.135
Professionals	56	27.59	39	19.21	32	15.76	76	37.44		
Associate professionals	28	28.87	30	30.93	19	19.59	20	20.62		
Service workers	38	20.00	49	25.79	46	24.21	57	30.00		
Technicians	18	33.33	14	25.93	7	12.96	15	27.78		
Never employed	42	39.62	22	20.75	26	24.53	16	15.09		

*Notes*: LoF-LoQ = drinks less than one time a month and not more than 1 drink for women/2 drinks for men at one time, HiF-LoQ = drinks more than one time a month but not more than 1 drink for women/2 drinks for men at one time, LoF-HiQ = drinks less than one time a month but drinks more than 1 drink for women/2 drinks for men at one time, and HiF-HiQ = drinks more than one time a month and drinks more than 1 drink for women/2 drinks for men at one time. ** *p* < 0.001; * *p* < 0.05.

**Table 5:** Perception of drinking and acceptance of drinking by drinking groups.

	Low Frequency-Low Quantity (LoF-LoQ)		High Frequency-Low Quantity (HiF-LoQ)		Low Frequency-High Quantity (LoF-HiQ)		High Frequency-High Quantity (HiF-HiQ)		*F*	*f*
	Mean	SD	Mean	SD	Mean	SD	Mean	SD		
**Perceptions of drinking**										
*Item*										
one of life’s pleasures	3.21_a,b_	1.12	3.56_a,c_	0.98	3.00_c,d_	1.01	3.66_b,d_	0.89	15.88 **	0.264
drinking with someone is a way of showing friendship	3.74_e,f_	0.87	3.95_e_	0.78	3.77_g_	0.77	4.10_f,g_	0.64	10.35 **	0.195
drinking has no benefits	3.25	0.98	3.26	1.05	3.47	1.05	3.18	0.96	2.38	0.110
**Acceptance of drinking**										
*Component*										
With Young Children	1.46_h_	0.61	1.73_h,i_	0.69	1.43_i_	0.64	1.58	0.60	7.59 **	0.190
Adult Private Context	2.22	0.75	2.30	0.71	2.23	0.71	2.41	0.81	2.34	0.105
Social Drinking for Males	2.20_j,k_	0.55	2.40_j,l_	0.60	2.28_m_	0.56	2.62_k,l,m_	0.65	17.25 **	0.268
Social Drinking for Females	1.95_n_	0.55	2.09_o_	0.58	2.02_p_	0.62	2.28_n,o,p_	0.64	9.93 **	0.208

*Notes*: LoF-LoQ = drinks less than one time a month and not more than 1 drink for women/2 drinks for men at one time, HiF-LoQ = drinks more than one time a month but not more than 1 drink for women/2 drinks for men at one time, LoF-HiQ = drinks less than one time a month but drinks more than 1 drink for women/2 drinks for men at one time, and HiF-HiQ = drinks more than one time a month and drinks more than 1 drink for women/2 drinks for men at one time. Means sharing a common subscript are statistically different at α = 0.05 according to the Tukey HSD procedure. Perception of drinking scale: 1 = strongly disagree, 5 = strongly agree; Acceptance of drinking scale: 1 = should not drink; 2 = can drink a little but without any real effects, 3 = can drink and feel some effects but not get intoxicated, 4 = getting intoxicated sometimes is OK, 5 = getting intoxicated is always OK. ** *p* < 0.001.

**Table 6:** Effects of drinking and Reasons for drinking by drinking level groups.

	Low Frequency-Low Quantity (LoF-LoQ)		High Frequency-Low Quantity (HiF-LoQ)		Low Frequency-High Quantity (LoF-HiQ)		High Frequency-High Quantity (HiF-HiQ)		*F*	*f*
	Mean	SD	Mean	SD	Mean	SD	Mean	SD		
**Effects of drinking**										
*Component*										
Coping	2.29_a,b_	0.89	2.72_a_	0.96	2.50_c_	0.98	2.85_b,c_	1.00	12.17 **	0.223
Mood Enhancer	2.76_d,e_	0.87	3.03_d_	0.80	2.86_f_	0.88	3.26_e,f_	0.86	12.04 **	0.225
Problem Behavior	1.29_g_	0.48	1.37	0.52	1.33	0.52	1.44_g_	0.50	2.79 *	0.109
Sex	1.61_h,i_	0.75	2.23_h,j_	0.93	1.55_j,k_	0.88	2.07_i,k_	1.03	15.06 **	0.321
**Reasons for drinking**										
*Component*										
Psychological Reasons	1.95_l,m_	0.71	2.20_l_	0.65	2.09	0.74	2.26_m_	0.71	7.01 **	0.169
Social Reasons	2.59_n,o,p_	0.58	2.76_n_	0.47	2.78_o_	0.55	2.88_p_	0.56	8.85 **	0.186
Thirst/Taste	1.48_q_	0.50	1.59	0.61	1.54	0.53	1.64_q_	0.63	2.85 *	0.105

*Notes*: LoF-LoQ = drinks less than one time a month and not more than 1 drink for women/2 drinks for men at one time, HiF-LoQ = drinks more than one time a month but not more than 1 drink for women/2 drinks for men at one time, LoF-HiQ = drinks less than one time a month but drinks more than 1 drink for women/2 drinks for men at one time, and HiF-HiQ = drinks more than one time a month and drinks more than 1 drink for women/2 drinks for men at one time. Means sharing a common subscript are statistically different at α = 0.05 according to the Tukey HSD procedure. Effects of drinking scale: 1 = seldom, 5 = always; Reasons for drinking scale: 1 = not at all important, 4 = very important. ** *p* < 0.001; * *p* < 0.05.

**Table 7:** Reasons for not drinking by drinking groups.

	Low Frequency-Low Quantity (LoF-LoQ)		High Frequency-Low Quantity (HiF-LoQ)		Low Frequency-High Quantity (LoF-HiQ)		High Frequency-High Quantity (HiF-HiQ)		χ^2^	Cramer’s *V*
	*n*	%	*n*	%	*n*	%	*n*	%		
*Cluster*									5.26	0.068
Most reasons for not drinking are:										
not very important–unimportant (low-rating group)	63	29.86	45	21.33	44	20.85	59	27.96		
importance varied by reason (mid-range group)	66	25.48	67	25.87	52	20.08	74	28.57		
important–very important (high-rating group)	32	32.99	26	26.80	20	20.62	19	19.59		

*Notes*: LoF-LoQ = drinks less than one time a month and not more than 1 drink for women/2 drinks for men at one time, HiF-LoQ = drinks more than one time a month but not more than 1 drink for women/2 drinks for men at one time, LoF-HiQ = drinks less than one time a month but drinks more than 1 drink for women/2 drinks for men at one time, and HiF-HiQ = drinks more than one time a month and drinks more than 1 drink for women/2 drinks for men at one time.

## Supplementary Materials

The following are available online at http://www.mdpi.com/1660-4601/15/8/1675/s1, Table S1: Loadings for four-oblique components for “acceptance of drinking” (*N* = 1499), Table S2: Reasons for not drinking cluster description (*N* = 1221), Table S3: Loadings on three-oblique components for “effects of drinking” (*N* = 548), Table S4: Loadings on three-oblique components for “reasons for drinking” (*N* = 790).
