# Supplementary material for: Variables Associated with Alcohol Consumption and Abstinence among Young Adults in Central China
Source: Int J Environ Res Public Health. 2018 Aug 7;15(8):1675. doi: 10.3390/ijerph15081675 (PMC6121566; doi:10.3390/ijerph15081675)
Supplement: Supplementary file 1 [file ijerph-15-01675-s001.pdf]

International Journal of
Environmental Research and Public Health

MDPI

Table S1. Loadings on Four-Oblique Components for Acceptance of Drinking (N = 1499).

	Component 1	Component 2	Component 3	Component 4	Communality
	With Young Children	Adult Private Context	Social Drinking for Males	Social Drinking for Females	
Mother with young children	0.925				0.903
Father with young children	0.923				0.901
Man having dinner with partner		-0.959			0.941
Woman having dinner with partner		-0.954			0.941
Man at bar with friends			-0.895		0.855
Man out with colleagues			-0.752		0.790
Woman out with colleagues				0.927	0.884
Woman at bar with friends				0.793	0.819

Note. Loadings < 0.3 are suppressed.

Table S2. Reasons for Not Drinking Cluster Description (N = 1221).

	Cluster 1 Low-rating n = 342 mean	Cluster 2 Mid-range n = 522 mean	Cluster 3 High-rating n = 357 mean
Alcoholism	1.34	2.39	3.18
Upbringing	1.68	2.50	3.32
Others disapproval	1.67	2.51	3.27
Being too young	1.61	2.47	3.20
Cost	1.39	2.05	2.83
Previously hurt by others drinking	1.39	2.14	3.04
Religion	1.11	2.00	2.57
Effects on performance at work/school	2.09	2.77	3.54
Have seen bad examples	2.18	2.58	3.55
Do not like being affected by alcohol	2.26	2.46	3.41
Health	2.20	2.90	3.56
Taste	2.03	2.20	3.08
Not interested	2.24	2.38	3.34

Note. Interviewees ranked reasons for not drinking on a 4-point scale of importance to them: not at all important; not very important; important; very important.

Table S3. Loadings on Three-Oblique Components for Effects of Drinking (N = 548).

	Component 1 Coping	Component 2 Mood Enhancer	Component 3 Problem Behavior	Component 4 Sex	Communality
Feel easier to talk about feelings	0.855				0.817
Forget problems	0.837				0.768
Become friendly	0.724				0.681
Feel relaxed		0.967			0.890
Feel happy		0.936			0.890
Have fun	0.324	0.437			0.628
Trouble with police			0.797		0.749
Become aggressive			0.791		0.745
Do something regret			0.529	0.305	0.626
Feel more attractive				0.798	0.793
Sex more pleasurable				0.765	0.739

Note. Loadings < 0.3 are suppressed.

Table S4. Loadings on Three-Oblique Components for Reasons for Drinking (N = 790).

	Component 1 Psychological Reasons	Component 2 Social Reasons	Component 3 Thirst/Taste	Communality
Relax	0.934			0.825
Feel good	0.877			0.770
Forget worries	0.798			0.683
Inhibition	0.662			0.622
Sociability		0.857		0.627
Others drinking		0.653		0.546
Celebrate	0.300	0.550		0.489
Meal enjoyment	0.357	0.536		0.560
Thirst			0.949	0.825
Taste	0.331		0.583	0.612

Note. Loadings < 0.3 are suppressed.
